# Family Capital, Learning Engagement, and Students’ Higher Education Gains: An Empirical Study in Mainland China

**DOI:** 10.3390/ijerph182111571

**Published:** 2021-11-04

**Authors:** Shutao Wang, Cui Huang

**Affiliations:** 1 College of Education, Zhejiang University, Hangzhou 310058, China; wangshutao@zju.edu.cn; 2 School of Public Affairs, Zhejiang University, Hangzhou 310058, China

**Keywords:** economic capital, social capital, cultural capital, learning engagement, higher education gains

## Abstract

This study aimed to determine whether learning engagement plays a mediating effect on the relationship between family capital and students’ higher education gains in mainland China. We used family capital, learning engagement, and higher education gains as measures and analyzed data using a structural equation model. Data were collected from 1334 students at a Chinese university. The results show that family cultural capital had the most significant effect on students’ learning engagement, while economic capital also played a positive role, and social capital had no significant impact. Learning engagement played a mediating role in the relationship between cultural capital and higher education gains, as did the relationship between economic capital and higher education gains. However, learning engagement did not have a mediating effect on the relationship between social capital and higher education gains. Our results show that we should focus on the importance of students’ learning engagement, improve the cultural capital of disadvantaged groups, and provide financial support for students from low-income families.

## 1. Introduction

Family capital, usually including economic capital, social capital, and cultural capital, is an important measure of disparities between different social strata and is an important consideration when studying the educational achievements of students from different social backgrounds [1]. The status attainment model proposed by Blau and Duncan [2] suggests that the quantity of family-owned resources has a significant impact on the higher education gains of children. Bourdieu and Passeron [3] have also pointed out that education is an important but concealed channel for social reproduction. Studies indicate that family capital (comprising elements such as the father’s occupation, level of education, income, and place of birth) plays an important role in the higher education gains of the next generation [4]. The impact of family capital on students’ higher education gains cannot be denied, but it can be difficult to identify the specific mechanisms behind this impact. Previous studies have focused on the influence of family capital of people from different social strata on higher education enrolment opportunities, mainly in the following two ways: (1) by exploring the disparities of social strata and focusing on the ways in which the specific factors of economic capital, social capital, and cultural capital affect enrolment in higher education institutions [5]; and (2) by measuring the enrolment rates for higher education in each stratum and comparing the ability of people from different social strata to acquire higher education opportunities [6]. However, research in this area has not yet produced a model that can explain the specific mechanisms behind the influence of family capital on students’ higher education gains. A simple affirmation of the effect of family capital is not sufficient; we must also seek to understand the specific role, process, and mechanism of this effect.

Scholars have previously pointed out that family background does not affect the development of students directly, but instead, affects development through a series of indirect variables [7]. Learning engagement focuses on the amount of time and effort that students invest in studying, as well as the effective use of school resources and the perception and acquisition of external support [8]. These factors are directly related to higher education gains. Learning engagement is an important index of students’ learning performance since it has a direct and strong relationship with academic achievement and has been an important aspect of research in the field of education [9]. However, the following questions remain unsolved: How is learning engagement affected by family capital? How does learning engagement further translate into higher education gains? Does the family capitals factor play an important role even in the Chinese context, where the majority of the students typically enroll in universities that are far away from their hometowns?

Therefore, in this study, in order to determine whether learning engagement has a mediating effect on the relationship between family capital and students’ higher education gains, we obtained data from the National Survey of Student Engagement for undergraduate students in mainland China (NSSE-China) and used a structural equation model to test this impact mechanism. In this study, based on a detailed review of the existing research literature, we put forward our own research hypotheses. Based on the brief description of the research method and process, this study constructed a structural equation model to explore the influence mechanism between family capital, learning investment, and education acquisition and discusses here the conclusions of the research.

## 2. Literature Review and Hypotheses

### 2.1. Economic Capital, Learning Engagement, and Higher Education Gains

The resource conversion model suggests that a family’s economic resources can be transformed into the next generation’s educational opportunities, resulting in unequal intergenerational transference [10]. Coleman [11] believes that family economic capital can provide children with crucial material resources for educational achievement. International comparative studies have consistently demonstrated that a family’s economic status plays an important role in the educational achievements of its offspring [12]. Studies on education conducted in China show that the role of a family’s socioeconomic status has been increasing, from the perspective of personal education, since China’s economic reforms and opening up [13]. According to family investment theories, the socioeconomic status of the family reflects the basic conditions of economic capital and human capital in the family environment. Families with a higher socioeconomic status are able to invest more capital in the development of their children, thereby exerting a significant impact on the growth, mentality, attitudes, education path, and life choices of their children in the future [14].

Research has shown that family economic status is closely related to learning engagement and that students from families with lower socioeconomic status find it difficult to approach studying with a positive attitude [15]. Families with a higher socioeconomic status are more likely to provide students with better learning conditions and material incentives, while students from low-income families more often lack quality education opportunities, face greater family pressure, and lack comparative access to educational resources and experiences [16]. One study has also indicated that families with higher socioeconomic status will invest more in education and acquire more educational resources for their children than families with lower socioeconomic status [17]. In addition, some have argued that when a family has a higher level of income, it is easier to afford, choose, and provide better material conditions and beneficial educational resources for students in the family [18]. Learning engagement is regarded as an important prerequisite for improving student achievement and students’ learning experience, and therefore it serves as a crucial indicator of academic success [8]. There is clear evidence that student engagement measures are significantly and positively related to perceived success in learning [19].

Based on these research results, we propose the following hypotheses:

**Hypothesis** **1a** **(H1a).** *Family economic capital ha* *s a positive impact on higher education gains;*

**Hypothesis** **1b** **(H1b).** *Family economic capital has a positive impact on learning engagement;*

**Hypothesis** **1c** **(H1c).** *Learning engagement has a positive impact on higher education gains;*

**Hypothesis** **1d** **(H1d).** *Learning engagement plays a mediating effect on the relation between family economic capital and higher education gains.*

### 2.2. Social Capital, Learning Engagement, and Higher Education Gains

Bourdieu and Passeron [20] defined social capital as the collection of actual or potential resources that individuals can obtain through the institutional network of social relations. The amount of individual social capital depends on the size of a person’s network and the proportion of resources obtained by members in the network through their abilities [21]. People with greater social capital can access various benefits offered by society with greater ease. Bourdieu and Passeron [20] also believed that the social capital that parents pass on to their children can provide them with more and better opportunities for attaining a higher level of higher education gains, resulting in a covert method of social reproduction. The distribution of social capital among different classes or groups is uneven, and people with the lowest social status have less subjective desire to receive higher education than objective opportunities [22]. Perna and Titus [23] also found that minority students in the United States faced greater challenges in enrolling in universities. They explained this as an effect of poor learning due to insufficient economic and cultural capital, as well as insufficient resources in the social network of family members.

Although some studies indicate that social capital can positively predict a student’s academic performance [24], more and more studies have shown that a family’s external capital has no impact or a negative impact on a student’s academic achievement. For example, some scholars believe that the main function of external social capital represented by network resources is to provide children with better learning opportunities, rather than to directly improve students’ performance in school [25]. This viewpoint was supported by several studies that questioned the positive role of a family’s external social capital [26]. Some of those studies indicate that a father’s professional reputation can significantly improve his children’s chances of entering high school, but negatively affect his children’s academic achievement [26]. Other studies indicate that social capital has no significant impact on children’s academic performance [27]. In addition, some empirical studies indicate that the influence of social capital on academic performance is regulated by family resources. If there are more resources in the family that are conducive to the academic performance of young people, social capital has a greater role in promoting the academic performance of young people. On the contrary, the promotion of social capital is limited [28].

Based on the above literature review, we propose the following hypotheses:

**Hypothesis** **2a** **(H2a).** *Family social capital does not have a positive impact on higher education gains;*

**Hypothesis** **2b** **(H2b).** *Family social capital does not have a positive impact on learning engagement;*

**Hypothesis** **2c** **(H2c).** *Learning engagement does not play a mediating effect on the relation between family social capital oriented by network resources and higher education gains.*

### 2.3. Cultural Capital, Learning Engagement, and Higher Education Gains

Cultural capital refers to the language and cultural values inherited by individuals from their families in the process of socialization, serving to promote academic achievement [29]. Bourdieu and Passeron [30] argue that the process of education involves the process of accepting and inheriting cultural capital; thus, education is a means of enforcing and transferring cultural capital. In this sense, descendants of families with higher levels of education inherit an advantage in gaining access to higher education and attaining higher education gains [31]. With regard to the specific mechanisms of this impact, Bourdieu [4] argued that cultural capital could help improve cognitive ability and learning skills so that students are better able to grasp the school curriculum and to achieve better results in education. The standardization of courses with a hegemonic class discourse in modern education continually strengthens the legitimacy of an aristocratic cultural capital that is based on the culture of members from the more dominant socioeconomic class [32]. The evaluation of academic performance is also based on the culture of the dominant class [29], where teachers award higher academic performance assessments to students who better understand the cultural values inherited from those in the upper classes; these student are typically more proficient in the dominant language style, behavior, and habits of the dominant class, and are more competent in navigating the corresponding education system [33].

The impact of family cultural capital on academic achievement may also be realized by increasing students’ level of learning engagement. An increase in cultural capital leads to a corresponding increase in the level of investment in students’ cognitive, habitual, and behavioral practices in studying, and the full set of hobbies and knowledge from a family environment can create measurable differences among college students [30]. Families rich in cultural capital possess more cultural resources, a rich learning atmosphere, and aspirations or expectations regarding their children’s academic achievement, so that the children’s goals and aspirations are likely to be higher as well [34]. Parents who have a higher level of education tend to have higher expectations for their children’s academic achievement. They can interact better with their children, especially in terms of providing guidance for their homework and developing good habits. For example, the larger the family’s collection of books, the greater the advantage enjoyed by the family in creating a better cultural atmosphere, through which parents can subtly influence their children to acquire effective reading habits and invest more effort in their studies. When students invest more effort in learning, they experience these advantages: (1) they become more familiar with the homework and requirements of their courses; (2) they are more engaged in writing and using collaborative problem-solving methods; (3) they become better at getting feedback from faculty members; (4) they gain a better understanding of their own state of knowledge; (5) they have better control over complex problems; and (6) they find it easier to work with people with different perspectives hailing from different backgrounds when asked to complete assigned tasks, while simultaneously achieving greater higher education gains [35]. Therefore, it is not surprising that when parents with similar professional status and income are compared with one another, the cultural indicators of the family seem to have the greatest impact on the higher education gains of university students [36]. In this way, cultural capital is very closely related to education. The greater the student’s family cultural capital, the better the student’s performance in school, and the greater the chance of the student pursuing further studies [20].

Based on the review above, we propose a third set of hypotheses:

**Hypothesis** **3a** **(H3a).** *Family cultural capital has a positive impact on higher education gains;*

**Hypothesis** **3b** **(H3b).** *Family cultural capital has a positive impact on learning engagement;*

**Hypothesis** **3c** **(H3c).** *Learning engagement plays a mediating effect on the relation between family cultural capital and higher education gains.*

**Figure 1:**
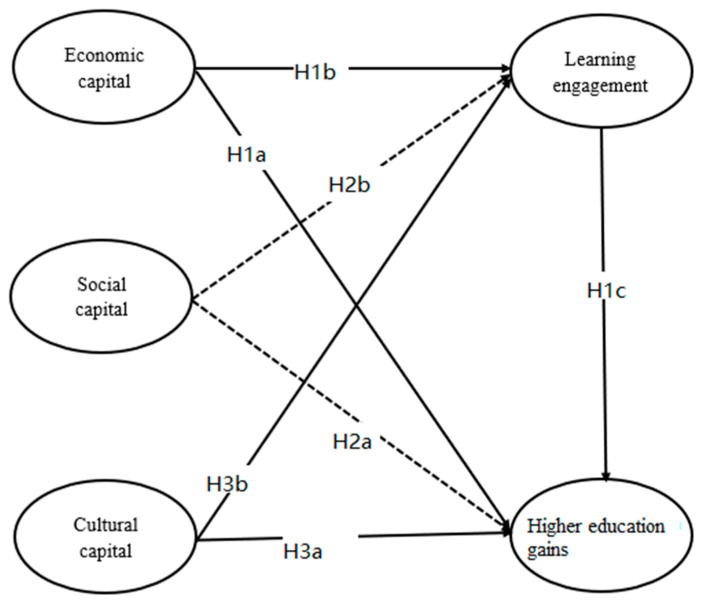
Analytical framework.

Through a summary of the literature, this paper uses the framework presented below for analysis (Figure 1):

## 3. Method

### 3.1. Sample and Data Collection

We sampled students according to the principle of equal proportion cluster random sampling from a total of nine different disciplines from a university in Shandong Province (Mainland China). Ten percent of the total number of students in each discipline was taken as the sample for this study. For example, if the total number of undergraduate students in a school was 120, then the sample in the education discipline was 12, and the valid sample was 9; if the total number of undergraduates in natural science was 4816, then the natural science sample taken in this study was 481, and the valid sample was 384 (Table 1). A total of 1600 questionnaires were administered to college students, and 1460 were returned, of which 1334 were valid. The students were informed that participation was voluntary and that there would be no negative effects if they refused or discontinued participation. Therefore, the number of responses varied across disciplines, with the highest number of responses from the sciences (n = 384, 28.8%), and the lowest number of responses from education (n = 9, 0.7%) (Table 1). The effective recovery rate was 83.38%. Of the students who participated, 48.1% (641) were male, and 51.9% (693) were female. The average age of the sample students was 21.03 ± 1.39, with ages ranging from 16 to 28. A total of 29.7% (396) of the students were first-year, 28.5% (380) were second-year, 29.1% (388) were third-year, and 12.7% (170) were fourth-year. The sample of the fourth-year students was smaller than the other groups because many of them were away from campus on internships or job interviews.

### 3.2. Measures

Data were collected using measures of family capital, learning engagement, and higher education gains.

Family capital. This variable mainly included economic capital, social capital, and cultural capital. (1) Economic capital refers to the family’s income level. In this study, we let sampled students report their father’s annual income and mother’s annual income. If the students had no father or mother, we asked them to fill in the income of other guardians. (2) Social capital refers to the family’s social network resources and its degree of access. We used father’s professional status and mother’s professional status to test family social capital. Social capital was divided into six categories, which were identified with a number between 1 and 6: officials of government or managers of enterprises and institutions, professionals, businessmen and service providers, industrial workers, farmers, and unemployed and others. (3) Cultural capital refers to the ability to acquire language and cultural behaviors from the family in ways that promote academic achievement, approximated by indicators such as the father’s level of education and the mother’s level of education. This variable was divided into 9 levels from “no formal education” to “doctoral education”. The fit statistics for the model of family capital (χ^2^/df = 4.886, NFI = 0.992, CFI = 0.994, IFI = 0.994, TLI = 0.982, RMSEA = 0.061) showed that the validity of this scale satisfied the statistical requirements.

Learning engagement [37]. This variable was measured using a model derived from the Indiana University NSSE to fit the characteristics of Chinese students and the domestic educational environment. NSSE is an annual project that conducts a survey of undergraduates at four-year colleges and universities to assess the extent to which they engage in a variety of educational practices associated with high levels of learning and development. Ever since its introduction in 2000, it has become a leading tool for assessing the quality of the undergraduate experience. It reflects the current international trend towards emphasizing a learner-centered education process and subjective evaluations of the value of education. Greater attention is paid to the internal mechanisms of higher education, including the following five major factors and 48 items: (1) degree of academic challenge, including 11 items, e.g., “I often read assigned textbooks or reference books”; (2) level of active collaborative learning, including 6 items, e.g., “I actively ask questions or participate in discussions in class”; (3) degree of teacher–student interaction, including 9 items, e.g., “I often discuss homework or grades with my teachers”; (4) diversity of educational experience, including 14 items, e.g., “I often communicate with people from different backgrounds”; and (5) on-campus support and resources, including 8 items, e.g., “I have a good relationship with my classmates”. A 4-point Likert scale was used to answer from never to very often. The construct validity fit indexes of the questionnaire (χ^2^/df = 5.0, RMSEA = 0.070, GFI = 0.82, NFI = 0.90, IFI = 0.90, CFI = 0.90) were relatively high, and the Cronbach’s α of the five factors were 0.71, 0.66, 0.86, 0.62, and 0.80, respectively. All these indicators confirm that the questionnaire had acceptable reliability and validity.

Higher education gains [17]. Higher education gains were not assessed by asking the students to report their grade point average (GPA) but by asking the students to report the improvement in their knowledge, skills, social values, and other abilities acquired in the process of receiving higher education. Higher education gains in this study were measured by ability improvement, which can better reflect students’ higher education gains in universities. The measure included 14 items: gains in cognitive ability, including 7 items, e.g., “Have you improved your critical thinking skills?”, and gains in practical ability, including 7 items, e.g., “Have you improved your organizational and leadership skills?” were the main two factors in our exploratory factor analysis. In this study, a 4-point Likert scale was used to answer from “no improvement” to “great improvement”. These factors showed a good fit with our operational definition of higher education gains. The fit statistics for this model (χ^2^/df = 3.300, NFI = 0.917, CFI = 0.940, IFI = 0.940, TLI = 0.924, RMSEA = 0.066) and its overall Cronbach’s α (0.887) showed that the validity and reliability of this scale satisfied the statistical requirements.

## 4. Results

### 4.1. Correlations between Family Capital, Learning Engagement, and Higher Education Gains

As Table 2 shows, the univariate correlation between economic capital and higher education gains was significant (r = 0.113, *p* < 0.01), followed by the univariate correlation between cultural capital and higher education gains (r = 0.146, *p* < 0.01); thus, Hypotheses 1a and 3a were supported. Similarly, a significant positive correlation was found between economic capital and learning engagement (r = 0.156, *p* < 0.01), followed by the correlation between cultural capital and learning engagement (r = 0.208, *p* < 0.01). These results further support Hypotheses 1b and 3b. In addition, there was a significant correlation between learning engagement and higher education gains (r = 0.623, *p* < 0.01); thus, Hypothesis 1c was supported.

However, the univariate correlation between social capital and higher education gains was not significant (r = 0.013, *p* < 0.01). Similarly, the correlation between social capital and learning engagement was not significant (r = 0.049, *p* < 0.01). Thus, both Hypotheses 2a and 2b were supported. Based on the stipulations of Wen, Hau, and Zhang [38] on the mediating test—i.e., if a variable is not relevant to the independent and dependent variable, it cannot be a mediating variable—our results supported Hypothesis 2c. Therefore, social capital was excluded from the structural equation model.

### 4.2. Mediating Effect of Learning Engagement on the Relationship between Economic Capital and Higher Education Gains, as Well as Culture Capital and Higher Education Gains

Hypotheses 1d and 3c were tested through a series of nested model comparisons, as shown in Table 3. Model 1, our baseline model, represented a fully mediating model. The paths from economic capital and cultural capital to learning engagement and from learning engagement to higher education gains were specified, respectively, but without direct paths from economic capital and cultural capital to higher education gains. All of the indexes in model 1 showed a good fit (χ^2^ /df = 4.142, RMSEA = 0.055, GFI = 0.971, NFI = 0.961, TLI = 0.959, CFI = 0.970) of the model. Against our baseline model, two nested models were tested. As shown in model 2, a direct path from economic capital to higher education gains was added. Model 3 was identical to model 1, except for the addition of two direct paths from economic capital and cultural capital to higher education gains, respectively. Model 1 was therefore nested within models 2 and 3. As shown in Table 3, the differences in the chi-square values were not significant for model 1, as compared with models 2 or 3. The good fits of models 2 and 3 were also not as good as model 1. Under the principle of model parsimony, these results suggest that model 1 best fitted our data.

**Figure 2:**
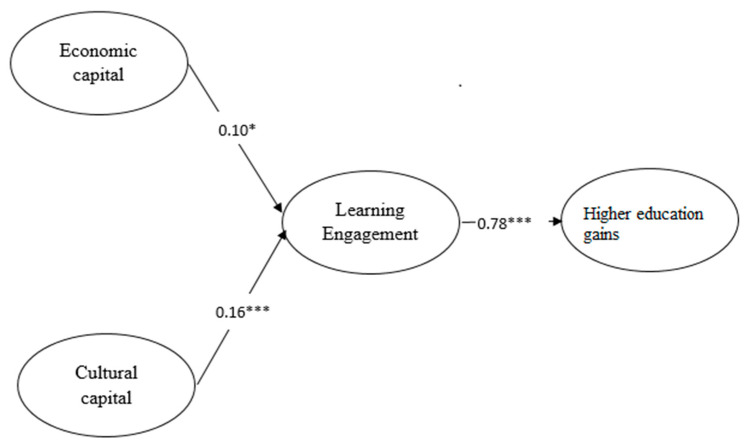
Mediating effect of learning engagement. * *p* < 0.05, *** *p* < 0.001.

Model 1, which is shown in Figure 2, indicated that both the coefficients from economic capital (β = 0.10, *p* < 0.05) and cultural capital (β = 0.16, *p* < 0.001) to learning engagement were significant, as was the coefficient from learning engagement to higher education gains (β = 0.78, *p* < 0.001). As shown in nested model 3, when leaning engagement came into play as a mediating factor between economic capital and higher education gains, as well as between cultural capital and higher education gains, the main effects of economic capital (β = −0.04, *p* > 0.05) and cultural capital (β = 0.03, *p* > 0.05) on higher education gains were not significant, which implies that learning engagement had full mediating effects between economic capital and higher education gains and between cultural capital and higher education gains. Thus, Hypotheses 1d and 3c were supported.

## 5. Discussion

We found that cultural capital had the most significant effect on learning engagement among the three types of family capital that were analyzed in this paper. The result is consistent with previous findings [39]. According to Bourdieu and Passeron’s [30] theory of cultural capital, students from upper-class families have relatively higher cultural capital and adapt better to the culture of university faculty members. They can respond to the communication signals made by teachers in a timely manner and are more likely to be influenced by educators. Therefore, their behavior affects how teachers grade their performance and ultimately aids them in achieving positive results [40]. At the same time, we found that students with high family cultural capital had better access to higher education resources and took greater advantage of high-quality higher education opportunities, such as access to study abroad [41], obtaining a minor degree, and enrolling in foreign language training. Other researchers have also found that educational inequality arises first because of the uneven distribution of cultural capital, followed by social capital and economic capital [42]. It is more effective to predict the impact that parents with better reading habits have on the gains of their children in school, especially when it comes to parents with lower levels of education [43]. Cultural capital is an internal intervention for students, and it is manifested mainly through the creation of a certain type of cultural atmosphere that entails the development of reading and other learning habits, such as learning-oriented motivation. The quality of such capital is sustainable, so that it plays a vital role in the absence of external interventions or supervision, which may be a reason why cultural capital had the most significant impact on learning engagement.

Though not as significant as the impact of cultural capital, economic capital also had a positive effect on students’ learning engagement. Research has consistently proved that the economic status of the family plays an important role in the higher education gains of the offspring [12]. The process of higher education offers incomparably rich resources and opportunities, as compared with basic education, and this access requires greater economic support from the family. If the economic conditions are strained, then the family can provide students only with the necessary school supplies, and can seldom afford to buy extracurricular books, allow students to travel abroad, or facilitate students’ participation in other activities that have been shown to be conducive to promoting the educational experience [18]. Accessing a double-major program, learning a foreign language, studying abroad, having interpersonal skills, and even participating in student groups and associations all strain the economic resources of students’ families. The current employment situation of university students is increasingly grim; returns on investment in higher education have been becoming worse for low-income groups, which will inevitably have a negative impact on the willingness of parents to invest in their children’s higher education. Insufficient financial support leads to a lack of investment in higher education for students with lower family economic capital, and the lack of motivation as well as the lack of educational opportunities and resource utilization further result in lower levels of higher education gains. These trends show that students from low-income family backgrounds should receive more assistance in order to compensate for their disadvantages.

Our analysis revealed that social capital oriented by network resources does not have a significant impact on students’ learning engagement. This result is supported by Zhao and Hong’s [25] study, but it is different from the results of previous studies [15]. One reason is that most previous studies on the impact of social capital on the learning experience have been based on basic education [44]. Our study was based on a survey of university students instead. The process of higher education is more complicated than that of basic education, particularly in its organizational structure, faculty composition, student training, and achievement evaluation. It is more difficult for family social capital to be involved in this process, compared with basic education. Another reason is that it may be related to the network resources-based social capital in this study. Unlike Coleman’s [11] closed social capital, which mainly comes from the social structure environment conducive to children’s growth, Bourdieu’s [20] network resource-based social capital comes from the social network of parents, and children are only indirect beneficiaries. Therefore, the main function of social capital represented by network resources is to provide children with better learning opportunities, rather than directly improving students’ performance in school, and social closure-based social capital can more directly affect good learning habits and strengthen the formation of learning abilities, thus more directly contributing to the improvement in students’ performance and grades [25].

Learning engagement plays a mediating effect on the relationship between cultural capital and higher education gains, as well as in the relationship between economic capital and higher education gains. This is in line with previous studies, which indicate that parents’ level of education and family income have a positive impact on the educational experience and academic development of their children [45]. Other studies have also found that students’ engagement is the crucial link between the classroom and personal background, which are essential contributors to the learning outcome [46]. This indicates that the promotion of students’ learning engagement improves the contribution of family capital. Therefore, it is recommended that we focus on students’ learning engagement.

There are some limitations in this study. First, this study used a cross-sectional methodology to determine the relation between family capital and higher education gains, which may not be able to assert a causal relationship between variables [47]. Second, the data of this study were obtained through the self-report method, which could also be collected through teacher report, parent report, and other methods.

## 6. Conclusions

Cultural capital is the strongest predictor of learning engagement and higher education gains among students. This means that on the one hand, when measuring students’ family background, the government and university not only should take family economic conditions as the main measurement index but also should take the amount of parents’ cultural capital as an important judgment basis. On the other hand, the government should attach importance to the popularization of education, especially basic education, in order to improve people’s education years, increase the acquisition of cultural capital among disadvantaged groups, and narrow the gap between the next generation and the previous generation in receiving education [39]. This form of family cultural capital can optimize students’ learning habits and improve their learning motivation and expectations. The goal is to make more effective use of educational resources, and ultimately, to enable students to succeed in higher education. In addition, students’ learning engagement should constitute a major focus of education development. When trying to reduce the negative influence of a disadvantaged family background, we should not ignore the role of the overall university environment and students’ own initiative in learning.

## Figures and Tables

**Table 1:** Number of samples from nine disciplines.

Disciplines	Number	Percent
Law	45	3.4%
Engineering	362	27.1%
Management	246	18.4%
Education	9	0.7%
Economics	64	4.8%
Science	384	28.8%
Agronomy	52	3.9%
Literature	154	11.5%
Medicine	18	1.3%

**Table 2:** Correlations among variables.

	M ± SD	Economic Capital	Social Capital	Cultural Capital	Learning Engagement	Higher Education Gains
Economic capital	5.40 ± 3.94	1.0				
Social capital	27,599.27 ± 43,834.83	0.302 ^**^	1.0			
Cultural capital	4.14 ± 2.19	0.324 ^**^	0.657 ^**^	1.0		
Learning engagement	3.20 ± 0.80	0.156 ^**^	0.049	0.208 ^**^	1.0	
Higher education gains	2.17 ± 0.49	0.113 ^**^	0.013	0.146 ^**^	0.623 ^**^	1.0

Note: ** *p* < 0.01.

**Table 3:** Goodness-of-fit index.

Model	$\chi^{2}$	df	$\Delta \chi^{2}$	$\chi^{2}$ /df	RMSEA	GFI	NFI	CFI	TLI
Model 1	165.661	40		4.142	0.055	0.971	0.961	0.970	0.959
Model 2	164.135	39	1.526	4.209	0.055	0.971	0.962	0.970	0.958
Model 3	163.444	38	2.217	4.301	0.056	0.971	0.962	0.970	0.957

Note: Model 1 = economic capital + cultural capital → learning engagement → higher education gains; Model 2 = economic capital + cultural capital → learning engagement → higher education gains and economic capital → higher education gains; Model 3 = economic capital + cultural capital → learning engagement → higher education gains and economic capital + cultural capital → higher education gains.

## Data Availability

Data are available from the corresponding author upon reasonable request.

## References

[1] Liu Z., Gao Y. (2011). Family capital, social stratification and attainment of higher education. J. High. Educ..

[2] Blau P.M., Duncan O.D. (1967). The American Occupational Structure.

[3] Bourdieu P., Eggleston J. (1974). The school as a conservative force: Scholastic and cultural inequalities. Contemporary Research in the Sociology of Education.

[4] He X. (2019). The Impact Analysis of Family’s Capital on Access to Higher Education. Forum Contemp. Educ..

[5] Dong J.Y. (2015). The tension between Chinese peasants’ family capital reproduction strategies and massification in higher education. Int. J. Parents Educ..

[6] Hu R., Zhang Y.Z. (2007). Stratification mobility rate of higher education opportunity and its impact factors. Tsinghua J. Educ..

[7] Bradley R.H., Corwyn R.F. (2002). Socioeconomic status and child development. Annu. Rev. Psychol..

[8] Wang S., Zhang D. (2020). Perceived teacher feedback and academic performance: The mediating effect of learning engagement and moderating effect of assessment characteristics. Assess. Eval. High. Educ..

[9] Johnson M.L., Sinatra G.M. (2013). Use of task-value instructional inductions for facilitating engagement and conceptual change. Contemp. Educ. Psychol..

[10] Li Y. (2006). Institutional change and educational inequality: Mechanisms in educational stratification in urban China (1966–2003). Soc. Sci. China.

[11] Coleman J.S. (1988). Social capital in the creation of human capital. Am. J. Sociol..

[12] Donald J., Treiman K.Y., Kohn M.L. (1989). Educational and occupational attainment in 21 countries. Cross-National Research in Sociology.

[13] Wu X.G. (2016). Higher education, elite formation and social stratification in contemporary China. Society.

[14] Matthews K.A., Gallo L.C. (2011). Psychological perspectives on pathways linking socioeconomic status and physical health. Annu. Rev. Psychol..

[15] Randolph K.A., Fraser M.W., Orthner D.K. (2006). A strategy for assessing the impact of time varying family risk factors on high school dropout. J. Fam. Issues.

[16] Cuthill M. (2010). Engaged outreach: Using community engagement to facilitate access to higher education for people from low socio-economic backgrounds. High. Educ. Res. Dev..

[17] Shi L.S., Chen Y.m., Hou X., Gao F.Q. (2013). Social economic status and study engagement: The mediating effects of academic self-efficacy among junior high school students. Psychol. Dev. Educ..

[18] Villar A., Hernàndez F.J. (2015). The expenditures on higher education in times of crisis. Analysis of the behavior of the family investment according to social class in the European Union. Am. J. Educ. Res..

[19] Zilvinskis J., Masseria A.A., Pike G.R. (2017). Student engagement and student learning: Examining the convergent and discriminant validity of the revised national survey of student engagement. Res. High. Educ..

[20] Bourdieu P., Passeron J.C. (1977). Reproduction in Education, Society and Culture.

[21] Burt R.S. (2000). The network structure of social capital. Res. Organ. Behav..

[22] Bourdieu P., Passeron J.C. (2002). Heir: College Students and Culture.

[23] Perna L.W., Titus M.A. (2005). The relationship between parental involvement as social capital and college enrolment: An examination of racial/ethnic group differences. J. High. Educ..

[24] Liu B., Zhang Y., Li J. (2015). Family SES and adolescent educational expectation: Mediating role of parental involvement. Peking Univ. Educ. Rev..

[25] Zhao Y.D., Hong Y.B. (2012). Social capital and educational gains. Sociol. Res..

[26] Yang Y., Yu X., Liu Z. (2014). Initial endowment, social capital, and high school students’ achievement. Tsinghua J. Educ..

[27] Li X., Zheng L. (2016). Does social capital matter? The influence of family intergenerational closure on student academic performance in rural China. J. Educ. Stud..

[28] Tian F., Jing Y. (2018). Family class background, social capital and adolescent academic achievement. Fudan J..

[29] De Graaf N.D., De Graaf P.M., Kraaykaamp G. (2000). Parental cultural capital and educational gains in the Netherlands: A refinement of the cultural capital perspective. Sociol. Educ..

[30] Bourdieu P., Passeron J.C. (1979). The Inheritors: French Students and Their Relation to Culture.

[31] Katsillis J., Rubinson R. (1990). Cultural capital, student achievement, and educational reproduction: The case of Greece. Am. Sociol. Rev..

[32] Connell R.W. (2002). Making the difference. Discourse Stud. Cult. Politics Educ..

[33] Sullivan A. (2001). Cultural capital and educational gains. Sociology.

[34] Pitman T. (2013). ‘Miraculous exceptions’: What can autobiography tell us about why some disadvantaged students succeed in higher education?. High. Educ. Res. Dev..

[35] Kuh G.D. (2009). The national survey of student engagement: Conceptual and empirical foundations. New Dir. Inst. Res..

[36] Low R.Y.S. (2015). Raised parental expectations towards higher education and the double bind. High. Educ. Res. Dev..

[37] Tu D.B., Shi J.H., Guo F.F. (2013). A metric study on NSSE-China. Fudan Educ. Forum.

[38] Wen Z.L., Hau K.T., Zhang L. (2005). A comparison of moderator and mediator and their applications. Acta Psychol. Sin..

[39] Guo C., Min W. (2006). The effect of familial economic and cultural capital on educational attainment in China. J. High. Educ..

[40] Dimaggio P. (1982). Cultural capital and school success: The impact of status culture participation on the grade of U.S. high school students. Am. Sociol. Rev..

[41] Green W., Gannaway D., Sheppard K., Jamarani M. (2015). What’s in their baggage? The cultural and social capital of Australian students preparing to study abroad. High. Educ. Res. Dev..

[42] Hanley J.E., McKeever M. (1997). The persistence of educational inequalities in state-socialist hungary: Trajectory-maintenance versus counter selection. Sociol. Educ..

[43] Cook C.J. (1997). Cultural Practices and Socioeconomic Attainment: The Australian Experience.

[44] Lindfors P., Minkkinen J., Rimpelä A., Hotulainen R. (2018). Family and school social capital, school burnout and academic achievement: A multilevel longitudinal analysis among Finnish pupils. Int. J. Adolesc. Youth.

[45] Ackerman B.P., Brown E.D., Izard C.E. (2004). The relations between persistent poverty and contextual risk and children’s behavior in elementary school. Dev. Psychol..

[46] Zepke N. (2015). Student engagement research: Thinking beyond the mainstream. High. Educ. Res. Dev..

[47] Wang S., Zhang D. (2020). The impact of perceived social support on students’ pathological internet use: The mediating effect of perceived personal discrimination and moderating effect of emotional intelligence. Comput. Hum. Behav..

